# Velpeau

**Published:** 1854-08

**Authors:** 


VELPEAU.—Here is a true veteran for you; here is a surgeon with the three requisites—a lady's hand, an eagle's eye and a lion's heart; here is the man who enjoys the "*digitis monstror præterientium:*" you see, at last, him that you have been hearing of all your life, and before you ever thought of medicine; you have, perhaps, heard your cousin or your friend, who was in Paris years before you, speak of seeing Velpeau cut off an arm, or a hand, on such and such a morning, and you yourself also watch him do the same thing, on the same spot, fifty times; in fact, you see him perform two or three operations almost every morning you take the trouble to be at his amphitheatre at La Charité. He will cut for stone, operate two or three times for cross-eye, take out tumours from the breasts of two or three women, amputate a finger, a thumb or a leg, almost any morning you choose to name, after he has visited these long lines of beds up stairs and down stairs, in male and female wards, and after he has lectured an hour, and all before breakfast. Heaven knows at what hour he has risen!—what he has not written in one of the works he is publishing, on the "*Maladies du Sein et de la Region Mammaire,*" or some other. But I do know that he has not yet finished, because, before his carriage and pair leave the Rue Jacob, in front of La Charité, and when you have gone to the Café de Paris, to Madame Dijon's, to 66 Rue de Seine, or with me to my rooms on the garden of the Luxembourg, *he* has remained to prescribe for a score of what are called out-door patients, whom he examines as fast as they can be marshalled in a room on the ground floor. Then M. Velpeau's private consultations at his own residence, and his visits, have not yet commenced. But notwithstanding, you will sometimes meet him on the *Pont Neuf*, or the *Pont de Carrousél*, with a young lady on his arm, as like as father and daughter can be, as they walk to the Louvre or the garden of the Tuilleries. And you ask again is this man, strolling quietly by, the hero of a hundred bloody operations, the creator of bold systems, and originator of new modes of practice—the bold generalizer, who has laid a master hand on almost every department of medicine; who has written a hundred volumes, whom everybody quotes, even men who study specialities, and who recognize that, during a life of ceaseless toil and exertion, *he* has made a speciality of every department of his profession, and has rendered himself as competent to pronounce upon their respective merits as any enthusiast of a single one, whether it be in surgery, materia medica, obsterics, physiology, diseases of the eye, or anything you please to name. M. Velpeau is a bold, successful innovator in each and every one, and he has stamped his name in every text book that the student uses. He has written, and now writes, tomes filled with costly illustrations, which the medical world reads and consults, and swear at, sometimes, but always quote or follow. There are none so dauntless as not to do him reverence, none so bold but do not ask what Velpeau said this morning at a clinique. Does he discover anything, he goes to have Velpeau test, and make it go down. With Velpeau's sanction, he is confident against the world in arms, and goes forth with redoubled zeal. Does Velpeau damn the new project, it is like a dash of cold water, and the thermometer of his ardour falls instantly. I think that, generally, M. Velpeau, after his very large and most enlightened experience and observation, has a right to pronounce, and that he does so cautiously and with discrimination. He has, at times, been quick, and perhaps wrong, and now I think the microscopists will make him rue the day that he decided too dogmatically against their true and false cancers; for, already M. M. Lebert and Robin will show him that the glass is surer and more unerring than the unaided experience of even *his* eye and finger, and he will confess, or what is worse, posterity will do it for him, that true cancer and the unmalignant fibro plastic growths reveal themselves by signs, more certain than those gained by observation and sight simply. Perhaps, too, M. Ricord, or his successors, will demonstrate, that in proclaiming his doctrine of syphilization, a true advance has been made which time will sanction and enforce, and that its cardinal points are *real*, which M. Velpeau utterly repudiates and rejects *ab ovo*, root and branch. Velpeau must recollect that Ricord, though young and vigorous, resembles him in one respect—*he* does not take vacation either.* An old man, however great, is apt to remember triumphs won in youth from giants, and he grows too confident thereby. He is apt to forget that the tables turn, as age advances and years roll by, and when the conflict lies between the old and the youthful, but, at the same time, the vigorous, the cautious and the observant.

---

*M. Ricord informed me that, with the exception of the last, he had not indulged in the ordinary *vacance* of the Paris doctor, for twenty years. He is at the *Hospital du Midi* the year round.

As a lecturer, Velpeau is not remarkable, though he is sufficiently fluent and pleasant. Some new commers find his French easy to understand; but though he does not mumble his words, and splutter, as M. Roux did, yet there are several in Paris who speak very much plainer and better than he. I could cite Trousseau and Maisonneuve for examples. His tone is somewhat monotonous, and however enlivened by an occasional dry jest, yet there is neither much eloquence nor brilliancy. But Velpeau's attraction is his fame, his knowledge, and the perfect flood of light which his vast experience enables him to throw on every subject. You feel that he utters opinions which you can set down as rules and axioms for your guidance in practice. He speaks *ex cathedra* upon every topic; his words are golden, for they flow refined from a vast mass of material collected during years of earnest toil, a toil directed by an enlightened understanding, and guided by an acquaintance and comparison, with not simply the stores of the past, but with the improvement of modern research.—*Charl. Med. Jour. and Rev.*

